# Further Characterization of *Clostridium perfringens* Small Acid Soluble Protein-4 (Ssp4) Properties and Expression

**DOI:** 10.1371/journal.pone.0006249

**Published:** 2009-07-17

**Authors:** Jihong Li, Daniel Paredes-Sabja, Mahfuzur R. Sarker, Bruce A. McClane

**Affiliations:** 1 Department of Microbiology and Molecular Genetics, University of Pittsburgh School of Medicine, Pittsburgh, Pennsylvania, United States of America; 2 Department of Biomedical Sciences, College of Veterinary Medicine, Oregon State University, Corvallis, Oregon, United States of America; 3 Department of Microbiology, College of Science, Oregon State University, Corvallis, Oregon, United States of America; Charité-Universitätsmedizin Berlin, Germany

## Abstract

**Background:**

*Clostridium perfringens* type A food poisoning (FP) is usually caused by *C. perfringens* type A strains that carry a chromosomal enterotoxin gene (*cpe*) and produce spores with exceptional resistance against heat and nitrites. Previous studies showed that the extreme resistance of spores made by most FP strains is mediated, in large part, by a variant of small acid soluble protein 4 (Ssp4) that has Asp at residue 36; in contrast, the sensitive spores made by other *C. perfringens* type A isolates contain an Ssp4 variant with Gly at residue 36.

**Methodology/Principal Findings:**

The current study has further characterized Ssp4 properties and expression. Spores made by *cpe*-positive type C and D strains were found to contain the Ssp4 variant with Gly at residue 36 and were shown to be heat- and nitrite-sensitive; this finding may help to explain why *cpe*-positive type C and D isolates rarely cause food poisoning. Saturation mutagenesis indicated that both amino acid size and charge at Ssp4 residue 36 are important for DNA binding and for spore resistance. *C. perfringens* Ssp2 was shown to bind preferentially to GC-rich DNA on gel-shift assays, while Ssp4 preferred binding to AT-rich DNA sequences. Maximal spore heat and nitrite resistance required production of all four *C. perfringens* Ssps, indicating that these Ssps act cooperatively to protect the spore's DNA, perhaps by binding to different chromosomal sequences. The Ssp4 variant with Asp at residue 36 was also shown to facilitate exceptional spore survival at freezer and refrigerator temperatures. Finally, Ssp4 expression was shown to be dependent upon Spo0A, a master regulator.

**Conclusions/Significance:**

Collectively, these results provide additional support for the importance of Ssps, particularly the Ssp4 variant with Asp at residue 36, for the extreme spore resistance phenotype that likely contributes to *C. perfringens* type A food poisoning transmission.

## Introduction

*Clostridium perfringens*, a Gram-positive, anaerobic, sporeforming bacterium, can produce at least 17 different toxins. However, individual *C. perfringens* strains never produce this entire toxin repertoire. A commonly used system [1] exploits this variability in toxin production to classify individual *C. perfringens* isolates into types A–E, based upon their production of four typing toxins (alpha, beta, epsilon and iota toxins).

About 1–5% of type A isolates produce another toxin, named *C. perfringens* enterotoxin (CPE), which is responsible for causing the gastrointestinal (GI) symptoms of *C. perfringens* type A FP [2]. This FP currently ranks as the second most commonly reported bacterial foodborne disease in the USA and UK [3], [4], where (respectively) over 250,000 or 85,000 cases occur annually. Those cases usually resolve without long-term consequence, but *C. perfringens* type A FP can be fatal in the elderly or debilitated individuals. Consequently, *C. perfringens* ranks among the three or four most common bacterial causes of foodborne death [1], [3], [4]. In addition to FP, CPE-positive type A *C. perfringens* strains also cause about 5–15% of all cases of nonfoodborne human gastrointestinal diseases, including sporadic diarrhea and antibiotic-associated diarrhea [1], [2].

In type A isolates, the gene (*cpe*) encoding CPE can be either chromosomal or plasmid-borne [5], [6]. Most (∼75%) FP cases are caused by type A isolates carrying a chromosomal *cpe* gene (C-*cpe*) [5], [6], [7], [8], [9], [10]. Recent studies have provided at least three (possibly interrelated) explanations for this strong association between C-*cpe* isolates and FP. First, type A C-*cpe* isolates were found to be more prevalent than type A plasmid *cpe* (P-*cpe*) isolates in American retail meat products [11], which are important vehicles for *C. perfringens* type A FP [1]. Second, type A C-*cpe* isolates usually grow faster, and over a broader temperature range, than do type A P-*cpe* isolates [12], which should favor the multiplication of C-*cpe* isolates in foods so these bacteria can reach the food burden necessary for inducing disease. Finally, compared to the vegetative cells or (particularly) spores of type A P-*cpe* isolates, the cells/spores of type A C-*cpe* isolates were shown [12], [13], [14] to typically exhibit much more resistance against food safety-induced stresses such as heating, cold (refrigerator or freezer temperatures) storage, osmotic stress and food preservatives (e.g. nitrites). Since, i) spores of type A C-*cpe* isolates are present in retail foods [11] and ii) temperature abuse of foods during cooking or storage is the major underlying factor leading to *C. perfringens* type A FP outbreaks [1], the spore resistance phenotype of type A C-*cpe* isolates is likely to facilitate survival of these isolates in foods so they can later cause FP.

We recently identified [15] a major contributor to the exceptional spore resistance phenotype exhibited by the spores of most type A C-*cpe* isolates. Specifically, C-*cpe* isolates that produce resistant spores were found to express a variant of a novel small acid soluble protein named Ssp4. Whereas Gly is present at Ssp4 residue 36 in *C. perfringens* type A isolates producing sensitive spores, the Ssp4 residue 36 is an Asp in most, if not all, type A C-*cpe* isolates producing resistant spores. Inactivation of the gene (*ssp4*) encoding Ssp4 was shown to significantly increase the sensitivity of *C. perfringens* type A spores to both heat and nitrous acid (a fast-killing proxy assay for evaluating spore resistance against nitrite, an often used food preservative), directly demonstrating that Ssp4 plays an important role in spore resistance properties. Furthermore, when spores of those *ssp4* null mutants were complemented to express Ssp4 with an Asp at residue 36 (i.e., a Ssp4 Asp variant), they exhibited greater heat and nitrous acid resistance than did spores of the same mutant complemented to express Ssp4 with Gly at residue 36 (i.e., a Ssp4 Gly variant). This result proved that the Ssp4 Asp variant is an important contributor to the exceptional resistance phenotype exhibited by spores made by most type A C- *cpe* isolates. It was also shown that the exceptional protection afforded spores by the Ssp4 Asp variant apparently involves, at least in part, tighter spore DNA binding by this Ssp4 Asp variant, compared to the Ssp4 Gly variant made by most *C. perfringens* isolates [15].

The goal of our current study was to characterize further the contributions of the Ssp4 Asp variant to *C. perfringens* spore resistance properties and to begin examining how *C. perfringens* regulates expression of the *ssp4* gene during sporulation.

## Materials and Methods

### Bacterial strains and growth conditions

The bacterial strains and plasmids used in this study are listed in Table 1. All *C. perfringens* isolates were stored as stock cultures in Cooked Meat Medium (Sigma) at −20°C. The starter cultures were prepared from those stock cultures by overnight growth at 37°C in fluid thioglycolate broth (FTG) (Difco), as described previously [12], [15]. Sporulating cultures of *C. perfringens* were then prepared by inoculating 0.2 ml of the overnight FTG culture into 10 ml of Duncan-Strong (DS) sporulation medium [12]. After overnight incubation at 37°C, spores in the DS culture were purified as described previously [12]. Brain heart infusion (BHI) agar was used for plate count analyses [12].

**Table 1:** Bacterial strains and plasmids used in this study.

Strain or plasmid	Relevant characteristics	Sources or Refs
*C. perfringens*		
SM101	Food poisoning type A isolate, carries a chromosomal *cpe* gene	[18]
F4969	GI disease type A isolate, carries a plasmid borne *cpe* gene	[6]
CN1793	Type B isolate, toxigenic	UK, 1947
NCTC8533	Type B isolate, lamb dysentery	UK, 1950s
JGS1495	Type C isolate, porcine	unknown
CN5388	Type C isolate, human pigbel	unknown
CN4003	Type D isolate, lamb	unknown, 1956
JGS4138	Type D isolate, goat	USA, 2002
853	Type E isolate, calf with enteritis	North America
NCIB10748	Type E isolate, calf with enteritis	Institut Pasteur, reference strain
IH101	*spo0A* knock-out mutant derivative of SM101	[17]
SM101::*ssp4*	*ssp4* knock-out mutant derivative of SM101	[15]
F4969::*ssp4*	*ssp4* knock-out mutant derivative of F4969	[15]
*Plasmids*		
pDR81	*ssp2* fragment in the antisense direction to the *ssp2* promoter	[16]
pMRS123	*spo0A* ORF and ∼200 bp upstream sequence in pJIR751	[17]
pJIR751	*C. perfringens*/*E. coli* shuttle vector; Erm^r^	[17]
pCS	SM101 *ssp*4 ORF and ∼300 bp upstream sequence in pJIR751	[15]
pCF	F4969 *ssp*4 ORF and ∼300 bp upstream sequence in pJIR751	[15]
pD36E	pCS 36 amino acid site-directed mutagenesis D to E	This study
pD36N	pCS 36 amino acid site-directed mutagenesis D to N	This study
pD36K	pCS 36 amino acid site-directed mutagenesis D to K	This study

### Determination of the *ssp4* sequence in non-type A *C. perfringens* isolates

DNA was isolated from *C. perfringens* strains CN1794 (type B), NCTC8533 (type B), JGS1495 (type C), CN5388 (type C), CN4003 (type D), JGS4138 (type D), 853(type E) and NCIB10748 (type E) using the MasterPure gram-positive DNA purification kit (Epicentre). The primers ssp4proF and ssp4proR [15] were added (at a 5 µM final concentration) to a PCR mixture containing 1 µl of purified DNA template and 25 µl 2×Taq mixture (NEB), with a total volume of 50 µl. Each sample was then placed in a thermal cycler (Techne) and subjected to the following amplification conditions: 1 cycle of 95°C for 2 min, 35 cycles of 95°C for 30 s, 55°C for 40 s, and 68°C for 40 sec; and a single extension of 68°C for 5 min. The PCR products were cloned into a TOPO vector PCR2.1-TOPO (Invitrogen), which was sent for sequencing to the University of Pittsburgh Genomics Core Sequencing Facility. The *ssp4* genes sequences were deposited in GenBank under accession numbers GQ222061 (CN1793); GQ222062 (NCTC8533); GQ222063 (JGS1495); GQ222064 (CN5388); GQ222065 (CN4003); GQ222066 (JGS4138); GQ222067 (strain 853) and GQ222068 (NBIC107481).

### Measurement of spore resistance against heat and nitrous acid

The heat and nitrous acid resistance of spores were determined as described previously [13], [14]. Briefly, an aliquot of a DS spore culture was serially diluted in ddH_2_O, heated at 70°C for 20 min (to kill vegetative cells and promote spore germination), plated onto BHI agar, and incubated anaerobically overnight at 37°C to determine the initial Colony Forming Units (CFU)/ml of spores in the culture. Aliquots of the remaining DS culture were then heat-treated at 70°C for 20 min to kill vegetative cells, followed by a second heating at 100°C. At specified times, aliquots of those heated cultures were diluted and plated onto BHI agar. In other experiments, aliquots of DS cultures were heat-treated at 70°C for 20 min and then suspended in 100 mmol NaNO_2_, 100 mmol Na Acetate (pH 4.5) at room temperature for 1 h. Aliqouts of those nitrous acid-treated cultures were diluted and plated on BHI agar. After overnight anaerobic incubation (BD GasPack EZ Anaerobe Container systerm) at 37°C, the CFU/ml was counted and those results were used to calculate the decimal reduction times (D values), which is the treatment time needed to cause a 90% reduction in spore CFU/ml.

### Site-directed mutagenesis of the *ssp4* gene

Three site-directed mutations (D36K, D36E and D36N) were individually introduced into the *ssp4* gene that we had separately cloned [15] into both pJIR751 (a *C. perfringens-E. coli* shuttle plasmid encoding erythromycin-resistance (Em^r^)) and pTrcHis A (Invitrogen). Each of those mutations were generated using the QuikChange site-directed mutagenesis kit (Stratagene, La Jolla, CA). The reaction parameters were in accordance with the manufacturer's instructions. Each mutation was confirmed by DNA sequencing at the University of Pittsburgh Genomics Core Facility.

Mutated plasmid DNA resulting from each site-directed mutagenesis reaction was then transformed into XL1-Supercompetent Blue *E. coli*. The shuttle plasmids (named pJIR751-D36K, pJIR751-D36E and pJIR751-D36N) were separately electroporated into the SM101::*ssp4* null mutant. Transformants were selected on BHI plates containing 40 mg/L of Em. Spore heat and nitrous acid resistance were then determined, as described above.

pTrcHisA-D36K, pTrcHis-D36E and pTrcHis-D36N were separately transformed into *E. coli* DH5α. Transformants were selected by growth on LB containing Amp (50 mg/L). The presence of the desired mutated *ssp4* gene in each transformant was then confirmed by nucleotide sequencing. Overproduction and nickel affinity purification of each recombinant, His_6_-tagged rSsp4 mutant was performed as previously described [15]. The purified rSsp4 mutants were then used for Electomobility shift assay (EMSA) analyses as described later.

### Comparison of low temperature survival for spores made by wild-type SM101 or F4969, their isogenic *ssp4* null mutants, or complementing strains

The cold temperature (4°C or −20°C) resistance of spores produced by wild-type, *ssp4* null mutants, or complementing strains of those mutants were determined as described previously [12]. Briefly, sporulating cultures were prepared for each isolate by overnight growth in DS medium. After determining the total number of spores present in an aliquot of each DS medium culture at the start (day 0) of the experiment, the remainder of the DS culture was divided into small tubes, half of which were incubated at 4°C and the other half at −20°C. Aliquots were removed from these small tubes after 6 months and surviving spore numbers (determined as described in a preceding section) were used to calculate the log reduction after each treatment.

### Transformation of pDR81 into wild-type SM101 and an isogenic *ssp4* mutant

Plasmid pDR81 [16], which encodes an *ssp2* antisense gene that can inhibit *ssp1*, *ssp2* and *ssp3* transcription [16], was introduced by electroporation [2] into wild-type SM101 or SM101::*ssp4*. Em^r^ (40 mg/L) transformants were then selected. The resultant SM101 and SM101::*ssp4* transformants were designated as SM101 (pDR81) and SM101::*ssp4* (pDR81), respectively.

Heat and nitrous acid resistance of spores made by SM101 (pDR81) and SM101::*ssp4* (pDR81) were determined as described earlier.

### Small acid soluble proteins (SASPs) extraction and Western blotting

To evaluate SASPs presence in spores, *C. perfringens* SASPs were extracted, as described previously [15], from 50 mg of dry washed spores produced by specified *C. perfringens* strains. The extracted proteins were subjected to SDS-PAGE and the separated proteins were then transferred onto a nitrocellulose membrane. The resultant blot was probed either with antiserum raised against recombinant *C. perfringens* Ssp4 [15] or with antiserum raised against a *B. subtilis* α/β-type SASP named SspC. This SspC antiserum has been shown previously to cross-react with *C. perfringens* Ssp1, Ssp2 and Ssp3 [16].

### Spo0A production

To evaluate Spo0A production, *C. perfringens* strains were grown for 8 h at 37°C in DS medium and those cultures were then sonicated until >95% of the cells had lysed. Each culture lysate was then analyzed for the presence of Spo0A by Western blot using an antibody specific for *B. subtilis* Spo0A [17].

### Electromobility shift assays (EMSA)

A 3′-biotin-labeled, AT-rich (72.8% AT) C. *perfringens* DNA sequence was prepared using a biotin 3′-end DNA labeling kit (Pierce) as described previously [15]. Similarly, a 3′-biotin-labeled, GC-rich probe was prepared consisting of a 55 bp sequence from a GC-rich (69.1% GC) *C. perfringens* genomic DNA sequence. For this purpose, the following two oliogonucleotides were synthesized (Integrated DNA Technologies, Coralville, IA): Label-D2 (5′CTGGCGACTCAGAAGGGGCTCGAACCCTCGACCTCCG-GCGTG-ACAGGCCGGCACT-3′) and Label-R2 (5′-AGTGCCGGCCTGTCACGCCGGAG-GTCGAGGGTTCGAGCCCCTTCTGAGTCGCCAG-3′) and 3′-biotin-end-labeled by the manufacturer's instructions using a biotin 3′-end DNA labeling kit (Pierce).

The AT-rich probe was used in a modified EMSA to compare the DNA binding of the three site-directed, His_6_-tagged rSsp4 mutants. AT-rich and GC-rich probes were used in a modified EMSA, as described previously [15], to compare rSsp4 and rSsp2 binding preferences. Briefly, 1 µl of probe was incubated with 50, 100 or 200 ng of purified His_6_-tagged rSsp at 37°C for 1 h. Bound rSsp was then fixed to the DNA probe by the addition of glutaraldehdye (final concentration of 0.01% (v/v)) by 15 min incubation at 37°C. Those mixtures were loaded into a 6% polyacrylamide gel and electrophoresed in 0.5×TBE (Tris-borate-EDTA) buffer at 4°C for 1 h. DNA-protein complexes were then transferred to a positive charge nylon membrane (Roche Applied Science), UV crosslinked and detected with a LightShift Chemiluminescent EMSA kit (Pierce).

## Results

### Comparison of the Ssp4 sequence and spore heat- and nitrite-resistance properties amongst non-type A *C. perfringens* strains

Our initial Ssp4 study [15] found that the Ssp4 protein produced by 13 different type A strains shares an identical sequence, except for variations at amino acids 36 and 72. Those 13 type A isolates produced an Ssp4 with either Gly or Asp at residue 36 and either Asn or Lys at residue 72. As described in the Introduction, the presence of Asp at Ssp4 residue 36 was shown to be important for helping to mediate the exceptional spore resistance properties exhibited by most type A C-*cpe* FP isolates.

To further evaluate Ssp4 sequence diversity amongst *C. perfringens* isolates, the current study sequenced the *ssp4* ORF carried by eight strains belonging to *C. perfringens* type B, C, D or E (Tables 1 and 2). Those analyses revealed the presence of an identical *ssp4* ORF in all eight surveyed non-type A isolates. Furthermore, the *ssp4* ORF sequence present in these eight type B, C, D and E isolates identically matched the *ssp4* sequence found in type A isolates (e.g. F4969) producing an Ssp4 with Gly present at residue 36 and Lys present at residue 72.

**Table 2:** *Ssp4* sequence and spore resistance in various *C. perfringens* types.

Strain	Types	Toxin gene	3 6 res	72 res	Heat resistance (min)	Chemical resistance (log reduction)
SM101	A (FP)	*cpe* (chrom)	D	N	59.1±1.3	1.1±0.4
F4969	A (NFP)	*cpe* (plasmid)	G	K	0.5±0.0	4.0±0.5
CN1794	B	*cpb, etx, cpb2*	G	K	ND	ND
NCTC8533	B	*cpb, etx, cpb2*	G	K	1.4±0.5	4.2±0.7
JGS1495	C	*cpb, cpb2*	G	K	NA	NA
CN5388	C	*cpb, cpe, cpb2*	G	K	2.3±0.3	4.0±0.1
CN4003	D	*etx, cpe, cpb2*	G	K	ND	ND
JGS4138	D	*etx, cpe, cpb2*	G	K	2.7±0.6	5.3±0.1
853	E	*iota, cpe*	G	K	ND	ND
NBIC10748	E	*iota, cpe, cpb2*	G	K	ND	ND

The presence of the same *ssp4* sequence in F4969 and the eight surveyed non-type A isolates suggested that the spores produced by non-type A isolates might resemble the spores made by F4969 in terms of their heat- and nitrous acid-sensitivity. This hypothesis was tested by phenotyping the spores produced by a type B, C and D isolate for their ability to withstand boiling and nitrous acid (no type E isolate in our collection produced suitable levels of spores to conduct phenotype analyses). Results of those experiments showed that, relative to the resistant spores made by most type A C-*cpe* FP isolates (e.g. SM101), the spores of the three tested non-type A isolates of *C. perfringens* exhibited significantly more sensitivity to heat and nitrous acid (Table 2). Furthermore, the resistance properties determined for spores made by non-type A isolates closely matched those of spores produced by P-*cpe* isolates (e.g. F4969) (Table 2).

### Saturation mutagenesis of the SM101 *ssp4* gene at the codon encoding Asp residue 36 and phenotyping the spore resistance properties of those Ssp4 mutants

The Table 2 results supported previous results [15] demonstrating that variations at Ssp4 residue 36 are important for the heat- and nitrous acid-resistance properties of *C. perfringens* spores. Specifically, those C-*cpe* isolates (e.g. SM101, a transformable derivative of FP isolate NCTC 8798 [18]) forming exceptionally resistant spores make an Ssp4 with Asp at residue 36, while this Ssp4 residue is Gly in heat- and nitrous acid-sensitive *C. perfringnes* spores, including F4969 and the non-type A isolates phenotyped in Table 2.

To evaluate which amino acid properties at Ssp4 residue 36 are important for helping to mediate the exceptional resistance phenotype of spores produced by most C-*cpe* FP isolates, site-directed mutagenesis was performed on the SM101 *ssp4* gene cloned into either the pJIR751 shuttle plasmid (to allow testing of spore phenotypes) or the pTrcHis plasmid (to allow testing of DNA binding properties of rSsp4 mutants, which can be easily purified by nickel affinity chromatography due to their N-terminal, vector-encoded His_6_ sequence). These mutagenesis reactions created Ssp4 or rSsp4 variants where the natural Asp (D) present at residue 36 of the SM101 Ssp4 had been replaced by Glu (E), Lysine (K) or Asn (N).

**Figure 1:**
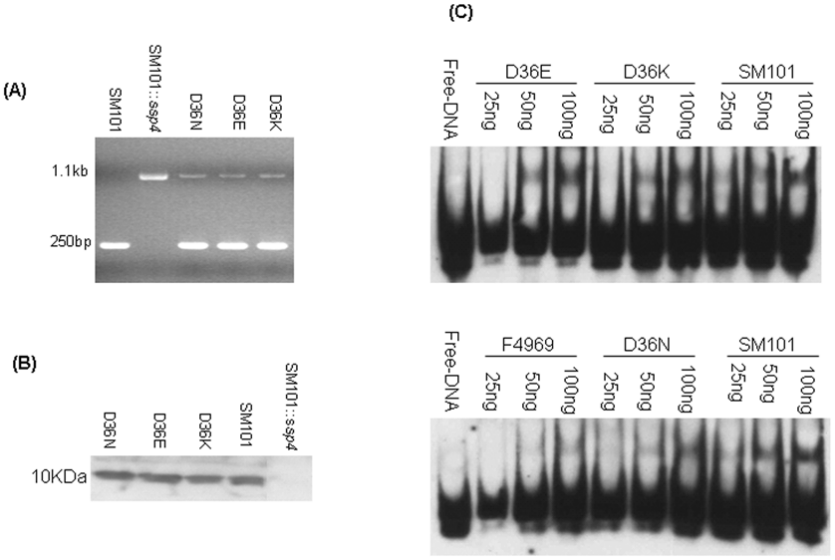
Site-directed mutagenesis of SM101 Ssp4 at residue 36 to change the natural codon encoding Asp (D) to encode Glu (E), Lysine (K) or Asn (N). (A) *Ssp4* ORF PCR results for: wild-type SM101; SM101::*ssp4*, SM101::*ssp4* transformed with pJIR751 carrying a mutant *ssp4* encoding one of the three site-directed Ssp4 mutants, i.e., D36N, D36E or D36K; (B) Western blot confirmation of expression of the Ssp4 D36N, D36E and D36K mutants. (C) DNA binding properties of purified recombinant His_6_-tagged rSsp4_D36E_, rSsp4_D36K_ or rSsp4_D36N_ and wild-type rSsp4 from SM101 or F4969. Top panel: Lane 1, free biotin-labeled *C. perfringens* DNA; lanes 8–10, indicated amounts of SM101 wild-type rSsp4 incubated with *C. perfringens* biotin-labeled DNA; lanes 2–4, indicated amounts of rSsp4_D36E_ incubated with *C. perfringens* biotin-labeled DNA; lanes 5–7, indicated amounts of rSsp4_D36K_ incubated with *C. perfringens* biotin-labeled DNA. Bottom panel: Lane 1, free biotin-labeled *C. perfringens* DNA lanes 2–4, indicated amounts of F4969 wild-type rSsp4 incubated with *C. perfringens* biotin-labeled DNA; lanes 5–7, indicated amounts of rSsp4_D36N_ incubated with *C. perfringens* biotin-labeled DNA.

The pTrcHis plasmids carrying each rSsp4 mutant were separately transformed into *E. coli*, while the pJIR751 plasmids carrying each mutant *ssp4* gene were separately transformed into a previously-created SM101 *ssp4* null mutant (SM101::*ssp4*) [15]. The presence in each transformant of a plasmid carrying mutated *ssp4* sequences was demonstrated by PCR (Fig. 1A) and the presence of the desired *ssp4* ORF mutation in the transformant was confirmed by sequencing (not shown). Production of each Ssp4 mutant (Fig. 1B) or rSsp4 mutant (not shown) was demonstrated by Ssp4 Western blotting.

Experiments were then performed to evaluate the resistance properties of spores produced by SM101::*ssp4* mutants after those null mutants had been complemented to express each Ssp4 variant with a residue 36 mutation. Control phenotypic comparisons first confirmed our previous observations [15] that inactivating the *ssp4* gene in SM101 reduced spore heat and nitrous acid resistance properties (Table 3). As we had also reported previously [15], complementation of the SM101::*ssp4* mutant to enable expression of Ssp4 with a wild-type Asp at residue 36 (SM101::*ssp4*-pCS) resulted in spores exhibiting exceptional resistant to both heat and nitrous acid. Similar complementation of this SM101::*ssp4* mutant so it expressed wild-type F4969 Ssp4 with a Gly at residue 36 (SM101::*ssp4*-pCF) resulted in a much more limited increase in spore heat and nitrous acid resistance properties.

**Table 3:** Heat and chemical resistance of SM101 transformants producing site-directed mutants.

	SM101	SM101::*ssp4*	SM101::*ssp4*- pCS	SM101::*ssp4*- pCF	SM101::*ssp4* -pD36E	SM101::*ssp4*- -pD36N	SM101::*ssp4* -pD36K
Heat resistance (D value) (min)	59.1±1.3	8.7±1.9	44.7±1.8	16.4±0.6	41.9±2.6	24.0±1.7	40.8±1.3
Chemical Resistance (log reduction)	1.1±0.4	4.0±0.1	1.1±0.6	3.2±0.1	1.2±0.3	2.2±0.2	1.6±0.2

When the resistance properties of spores made by SM101::*ssp4* transformants expressing the Ssp4_D36E_ or Ssp4_D36K_ mutants were tested (Table 3), those spores exhibited similar heat and nitrous acid resistance properties as SM101::*ssp4*-pCS spores containing the wild-type Ssp4 Asp variant. However, the spores produced by SM101::*ssp4* transformants expressing the Ssp4_D36N_ mutant had lower resistance against both heat and nitrous acid. Spores containing the Ssp4_D36N_ mutant exhibited similar resistance properties as spores made by SM101::*ssp4* transformed to produce the wild-type F4969 Ssp4 Gly variant.

To assess whether the resistance properties of spores containing Ssp4 with a site-directed mutation at residue 36 correlated with the DNA binding properties of their Ssp4 variant, wild-type SM101 rSsp4, the SM101 rSsp4 site-directed mutants, and wild-type F4969 rSsp4 were each purified and tested (Fig. 1C) for their DNA binding properties using an EMSA assay. Control EMSA analyses confirmed our previous report [15] that wild-type SM101 rSsp4 (with an Asp at residue 36) binds strongly to an AT-rich DNA probe, while F4969 rSsp4 (with a Gly at residue 36) binds less well to this DNA probe (Fig. 1C). Amongst the three SM101 rSsp4 variants with a mutated residue 36 amino acid, as created by site-directed mutagenesis for this study, Ssp4_D36E_ and Ssp4_D36K_ exhibited similar binding to the AT-rich DNA probe as did native SM101 rSsp4 with an Asp at residue 36, while Ssp4_D36N_ exhibited weaker DNA binding.

### Ssp4 preferentially binds to AT-Rich DNA

Results from Fig. 1 and our previous study [15] have demonstrated that Ssp4 can bind to an AT-rich probe mimicking *C. perfringens* DNA, which has a high (∼72%) overall AT content. Interestingly, the small acid soluble protein named SspC made by *Bacillus* spp. (another Gram-positive, sporeforming bacteria with low-GC% DNA) reportedly binds better to GC-rich DNA compared to AT-rich DNA [19], [20].

**Figure 2:**
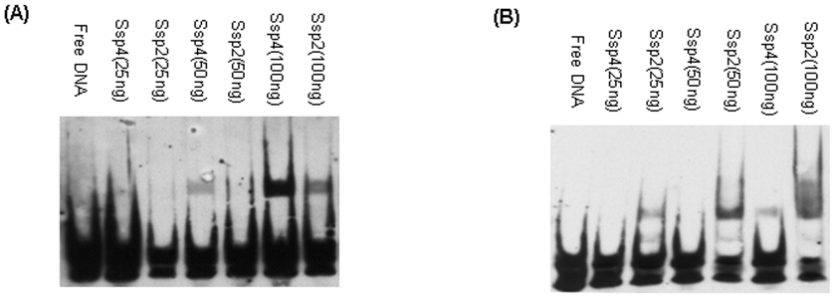
EMSA analysis of purified SM101 rSsp4 and rSsp2 binding to *C. perfringens* AT-rich biotin-labeled DNA (A) or CG-rich biotin-labeled DNA (B). Free DNA, biotin-labeled *C. perfringens* DNA (no rSsp); Lane 2–6, 25–100 ng of rSsp4 or rSsp2, as indicated, incubated with a AT-rich (Panel A) or GC-rich (panel B) *C. perfringens* biotin-labeled DNA probe.

Therefore, the current study performed an EMSA analysis to compare the binding of *C. perfringens* SM101 rSsp4 to AT-rich vs. GC-rich DNA probes. The current study also examined the binding of *C. perfringens* SM101 rSsp2 (which has a very similar sequence to *Bacillus* Ssp, as well as *C. perfringens* Ssp1 and Ssp3) to the same AT-rich vs. GC-rich DNA probes. These EMSA analyses revealed (Fig. 2) that purified rSsp2 binds preferentially to a probe containing a GC-rich (69.1% GC) sequence of *C. perfringens* DNA vs. a probe containing an AT-rich (72.8% AT) sequence of *C. perfringens* DNA. In contrast, purified rSsp4 exhibited a binding preference for the AT-rich vs. the GC-rich *C. perfringens* DNA sequence (Fig. 2).

### Ssp4 works in concert with other small acid soluble proteins (Ssp1, Ssp2, Ssp3) for maximal protection of *C. perfringens* spores against heat and nitrous acid

**Figure 3:**
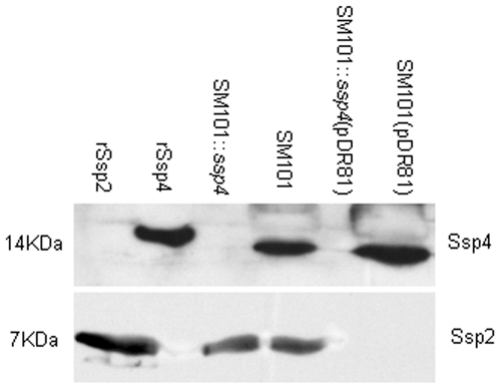
Western blot analyses of purified rSsp2 or rSsp4 (10 ng) wild-type SM101, an SM101 s*sp4* null mutant (SM101::*ssp4*), a wild-type SM101 transformant carrying the pDR81 plasmid encoding antisense RNA to inhibit production of Ssp1–3 (SM101(pDR81)) or the SM101 *ssp4* null mutant transformed to carry the pDR81 plasmid (SM101::*ssp4*(pDR81)). Top panel, Western blot probed with antiserum raised previously [15] against purified rSsp4; Bottom panel, Western blot probed with antiserum raised previously [16] against *B. subtilis* SspC, which is known to cross-react with *C. perfringens* Ssp1–3.

As mentioned above, *C. perfringens* produces at least four small acid soluble proteins. These include three proteins, named Ssp1–3, that differ from Ssp4 but share substantial sequence similarity with one another [16], [21], [22]. To explore why this bacterium produces so many different SASPs, an *ssp2* antisense plasmid was transformed into our SM101::*ssp4* null mutant. This antisense plasmid was shown previously to simultaneously block expression of Ssp1, Ssp2 and Ssp3 [16]. Consistent with those previous observations, Western blot analyses showed that neither SM101::*ssp4* (pDR81) nor SM101(pDR81) produced Ssp1, Ssp2 or Ssp3 proteins (Fig. 3), although SM101(pDR81) still produced Ssp4.

When heat and nitrous acid resistance were compared, wild-type SM101 spores exhibited about 7-fold higher resistance than spores of the *ssp4* null mutant (Table 4). Although the SM101(pDR81) transformant still produced Ssp4, its spores showed a reduced heat resistance compared against wild type spores, indicating that (in addition to Ssp4) Ssp1, Ssp2 and Ssp3 are also important for the full development of SM101 spore heat resistance. Importantly, SM101::*ssp4*(pDR81), which does not produce any of the four known *C. perfringens* Ssps, produced spores with virtually no heat resistance at 100°C.

**Table 4:** Heat and chemical resistance of SM101 or SM101::*ssp4* with or without the pDR81 antisense plasmid.

	SM101	SM101(pDR81)	SM101::*ssp4*	SM101::*ssp4* (pDR81)
Heat resistance D value (min)	59.1±1.3	18.1±2.7	8.7±1.9	1.4±0.1
Chemical Resistance (log reduction)	1.1±0.4	2.5±0.3	4.0±0.1	4.8±0.7

Spore nitrous acid resistance properties for these *C. perfringens* strains showed a similar pattern of differences as described above for spore heat resistance differences (Table 4). Spores of wild-type SM101 exhibited a nitrous acid-induced log reduction in viability of only 1.1, while nitrous acid caused a 4 log reduction in spore viability for SM101::*ssp4*, confirming a role for Ssp4 in SM101 spore nitrous acid resistance. Decreased production of Ssp1, Ssp2 and Ssp3 also reduced SM101 spore resistance against nitrous acid, although this 2.5 log reduction in spore viability was less than observed after *ssp4* gene inactivation. The strongest reduction in SM101 spore nitrous acid resistance (a 4.8 log reduction) was observed for SM101::*ssp4* (pDR81), which does not produce any of the known *C. perfringens* Ssps. Collectively, these results (Table 4) indicate that Ssp4 works in combination with the three other Ssps to protect *C. perfringens* spores against heat and nitrous acid treatment.

### Low temperature survival of spores produced by wild-type SM101 and F4969, their *ssp4* null mutants and complemented strains

As confirmed in Table 2, inactivation of the *ssp4* gene in SM101 or F4969 causes these isolates to produce spores with considerably less heat- and nitrous acid-resistance than their corresponding wild-type spores. In addition to heating and use of preservatives, storage of foods at low temperature (in refrigerators or freezers) is a very important food safety approach for controlling *C. perfringens* type A FP [12]. Therefore, the current study evaluated the involvement of Ssp4 for *C. perfringens* spore survival at 4°C and −20°C.

This study first confirmed previous conclusions [12] that wild-type SM101 spores exhibit exceptional survival at low temperatures, with only a 0.35 and 0.58 log reduction in spore viability measured after 6 months of storage at 4°C or −20°C, respectively (Table 5). Table 5 also shows, for the first time, that specific inactivation of the *ssp4* gene in SM101 reduced spore viability upon low temperature storage, with 0.82 or 1.91 log reduction in spore viability measured after 6-month storage of *ssp4* null mutant spores at 4°C or −20°C, respectively. These spore survival differences between wild type SM101 and its isogenic *ssp4* null mutant were statistically significant (P<0.01) at both 4°C and −20°C. Demonstrating that the decreased low temperature resistance of spores made by the SM101 *ssp4* null mutant was specifically due to inactivation of the *ssp4* gene, complementation of this mutant with a shuttle plasmid carrying the wild-type SM101 *ssp4* gene was able to substantially increase 6-month spore survival at both 4°C and −20°C. In contrast, transformation of the SM101::*ssp4* mutant with a shuttle plasmid carrying the wild-type F4969 *ssp4* gene more modestly increased 6-month spore survival of the SM101 *ssp4* null mutant upon storage at low temperatures.

**Table 5:** Cold resistance of wild-type, *ssp4* null mutants and complementing strains of SM101 and F4969.

Stains	4°C (log reduction after 6 month)	−20°C (log reduction after 6 month)
F4969	0.88±0.13	1.23±0.11
F4969::*ssp4*	2.02±0.15	3.12±0.10
F4969::*ssp4*(pCS)	0.70±0.10	1.01±0.33
F4969::*ssp4*(pCF)	1.30±0.20	1.81±0.10
F4969::*ssp4*(pJIR751)	1.82±0.10	2.70±0.40
SM101	0.35±0.10	0.58±0.17
SM101::*ssp4*	0.82±0.18	1.91±0.35
SM101::*ssp4*(pCS)	0.40±0.13	0.65±0.20
SM101::*ssp4*(pCF)	0.50±0.14	1.22±0.56
SM101::*ssp4*(pJIR751)	1.03±0.42	1.86±0.67

Consistent with previous observations, wild-type F4969 spores exhibited poorer survival at 4°C or −20°C compared to wild-type SM101 spores (Table 5). Table 5 also shows that inactivation of the *ssp4* gene in strain F4969 substantially decreased 6-month spore survival at both 4°C and −20°C. Spores of the F4969 *ssp4* null mutant exhibited much better 6-month survival at these low temperatures when they were complemented with a shuttle plasmid carrying the SM101 *ssp4* gene (Table 2). In contrast, complementation with the same shuttle plasmid carrying the wild-type F4969 *ssp4* gene caused a lesser increase in 6-month spore survival of the F4969 null mutant upon storage at either 4°C or −20°C.

Collectively, these results (Table 5) demonstrate that Ssp4 is important for spore survival at low temperatures and that the SM101 Ssp4 variant is better at protecting spores against low temperature-induced lethality than F4969.

### The role of Spo0A in regulating *ssp4* expression

Our previous studies [21], [22] have shown that production of Ssp1–3 requires a *C. perfringens* isolate to possess a functional *spo0A* gene. Therefore, the current study investigated whether Ssp4 expression is also Spo0A-dependent.

**Figure 4:**
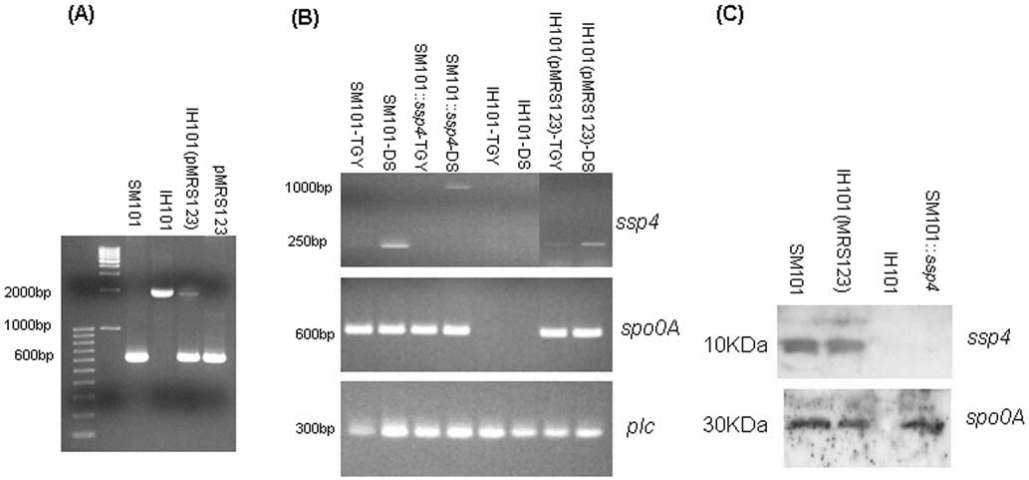
The role of Spo0A in regulation of Ssp4 production. (A) Total DNA isolated from SM101, *spo0A* null mutant IH101, and the complementing strain IH101(pMRS123), or plasmid pMRS123 alone, was subjected to PCR analysis using *spo0A*-specific internal primers CPP68 and CPP69 [17]. Note the larger size of the PCR product in IH101 due to insertional inactivation by chloramphenicol resistance gene *catP*. (B) Total RNA was isolated from SM101, SM101::*ssp4*, IH101 or IH101(pMRS123) grown as vegetative cultures in TGY or in DS sporulation-inducing medium, and then subjected to RT-PCR analysis using *ssp4*, *spo0A* or *plc* primers, as indicated. (C) Western blot analysis of Ssp4 and Spo0A production by SM101, IH101(MRS123), IH101 or SM101::*ssp4* grown in DS medium.

We first confirmed that *C. perfringens* IH101, a previously prepared *spo0A* null mutant of SM101 that cannot form spores [17], does not produce Spo0A. When Western blotting was used to compare by wild-type SM101 versus IH101 grown in Duncan-Strong (DS) sporulation medium (Fig. 4), the results obtained showed that DS cultures of SM101 and SM101::*ssp4* both produce Spo0A, but DS cultures of IH101 do not produce Spo0A. Confirming that this phenotype was specifically due to inactivation of the *spo0A* gene in IH101, complementation of IH101 with a plasmid carrying the wild-type *spo0A* gene restored Spo0A expression.

As expected from our previous studies [15], Ssp4 was produced by DS cultures of wild-type SM101 but not by SM101::*ssp4* [15]. As also reported previously [15], expression of Ssp4 by DS cultures of SM101::*ssp4* was restored by complementation with a shuttle plasmid carrying the wild-type *ssp4* gene. We now show that DS cultures of IH101 failed to produce Ssp4. However, DS cultures of the complementing strain IH101 (MRS123) did show Ssp4 expression, thereby demonstrating that Spo0A is specifically required for Ssp4 production.

## Discussion

*C. perfringens* FP isolates generally possess two complimentary virulence traits, i.e, production of CPE and the ability to form spores that are highly resistant to food environment stresses, such as heat, low temperatures and preservatives such as nitrites [1]. Since our previous study [15] had identified a novel Ssp4 variant as a major contributor to the spore resistance properties of FP isolates, the current study sought to better characterize Ssp4 proteins and to begin exploring the regulation of Ssp4 expression.

Our previous study [15] had determined that the *C. perfringens* type A FP isolates forming resistant spores usually produce an Ssp4 with Asp at residue 36, while other type A *C. perfringens* isolates, including both P-*cp*e isolates and *cpe*-negative isolates, typically make an Ssp4 with Gly at residue 36. Cross-complementation approaches with *ssp4* null mutants have also directly demonstrated that, 1) Ssp4 is an important mediator of spore resistance against heat and nitrous acid, and 2) the Ssp4 variant with Asp at residue 36 is better than the Ssp4 variant with Gly at residue 36 at protecting spores against heat and nitrites acid. In another previous study [12], we had shown that the spores of type A C-*cpe* isolates also exhibit exceptional survival at low temperatures, i.e., at 4°C and −20°C. The current study now reports that the strong low temperature resistance phenotype of spores made by FP isolates also involves Ssp4 and that the Ssp4 Asp variant is more against low temperature than the Ssp4 Gly variant. The exceptional low temperature resistance exhibited by spores containing the Ssp4 Asp variant is a likely contributor to *C. perfringens* FP transmission since meat and poultry products, common food vehicles for *C. perfringens* FP, are known to be contaminated with resistant spores of C-*cpe* isolates and are typically stored in refrigerators or freezers.

Determining that type A, C-*cpe* FP isolates typically produce spores whose resistance phenotype is mediated, in large part, by the Ssp4 Asp variant, while type A, P-*cpe* isolates produce spores whose sensitivity involves the Ssp4 Gly variant provided one explanation for the strong association between type A C-*cpe* isolates and FP. However, about 15% or 25% (respectively) of *C. perfringens* type C and D isolates also carry a *cpe* gene [23], [24] from which they produce similar amounts of an identical CPE protein as type A *cpe*-positive isolates. Interestingly, those *cpe-*positive type C or D isolates rarely, if ever, cause human FP. Results from the current study suggest that *cpe*-positive type C or D isolates may not commonly be involved in FP, at least in part, because they form spores that are sensitive to food environment stresses such as heating and preservatives. The *cpe*-positive type C and D isolates surveyed in this study also produce the same Ssp4 Gly variant as found in type A isolates producing sensitive spores. Since this same Ssp4 variant has been established as a major contributor to the sensitivity of type A isolates producing sensitive spores, the Ssp4 Gly variant is also likely to be an important factor behind the spore sensitivity of *cpe-*positive type C and D isolates, although this should be experimentally confirmed.

The current study also demonstrated that Ssp4 works in combination with Ssp1–3 to produce maximal spore resistance properties for *C. perfringens* type A C-*cpe* FP isolates. Furthermore, this work found that Ssp4 exhibits a preference for AT-rich DNA sequences, in contrast to Ssp2 (and most likely Ssp1 and 3 based upon their sequence similarities to Ssp2), which prefers binding to GC-rich DNA. This diversity in sequence binding preferences may help to explain why *C. perfringens* makes several Ssps. i.e., by producing multiple Ssps that bind to different chromosomal regions depending on their local AT% ratio, the entire chromosome can be maximally protected from damage induced by stresses such as heat or preservatives.

The properties of Ssp4 residue 36 that mediate spore resistance were also examined in the current study. These analyses revealed that spores retain exceptional heat and nitrous acid resistance if Lys or Glu were substituted for the Asp naturally found at Ssp4 residue 36 in *C. perfringens* type A FP isolates forming highly resistant spores. However, changing Ssp4 residue 36 from Asp to Asn produced sensitive spores resembling those made by isolates producing Ssp4 with Gly at residue 36. These results suggest that both the side chain length and presence of a charge at Ssp4 residue 36 may be important for mediating spore resistance properties. The strong resistance phenotype exhibited by spores carrying the mutant Ssp4 correlated with DNA binding properties of the corresponding purified rSsp4 mutant, supporting previous suggestions [15] that the DNA binding properties of Ssp4 variants are important determinants of spore resistance properties.

A bioinformatics search of Genbank revealed that other *Clostridium* spp. naturally carry ORFs encoding putative Ssp homologues with Glu (Cac4 of *Clostridium acetobutylicum* ATCC824, CnoI of *Clostridium noyvi* NT), Asp (Cac2 of *Clostridium acetobutylicum* ATCC824, Ssp2 of *C. perfringens* SM101, Cno2 of *Clostridium noyvi* NT, Cte2 of *Clostridium tetani*), Lys (Cac1 of *Clostridium noyvi* NT) or Gly (Ssp4 of *C. perfringens* ATCC3624) at the equivalent position as SM101 Ssp4 residue 36. The site-directed mutagenesis results of the current study might suggest that *C. acetylobutylicum* ATCC824, *C. novyi* NT and *C. tetani* E88 spores would exhibit substantial resistance against stresses such as heat, low temperature and food preservatives. However, this should be evaluated experimentally for two reasons. First, these isolates all carry ORFs encoding several different putative Ssps and it is clear from the current and previous studies [15] that no single Ssp fully determines spore resistance properties. Second, it is clear that Ssp4 variants are not the only contributor to variations in *C. perfringens* spore resistance properties. For example, the size of the spore core may also influence variations in *C. perfringens* spore resistance [25], [26]. It would also be of interest to evaluate whether other clostridial species exhibit intraspecies Ssp4 variants that influence spore resistance phenotypes, as occurs with *C. perfringens*.

Finally, the current results [22] revealed that, like Ssp1–3, expression of Ssp4 requires Spo0A. Spo0A is a master regulator of many genes expressed during sporulation [27] and late-stationary phase, so its involvement in Ssp4 expression is consistent with our previous finding that Ssp4 production is strongly sporulation-associated. Some Spo0A-regulated genes are regulated by Spo0A binding to sequences (0A boxes) located upstream of the ORF [27], [28]. A bioinformatics search detected potential 0A boxes upstream of all four *C. perfringens ssp* genes, including *ssp4*, in SM101. However, Ssp expression in *C. perfringens* may not only involve Spo0A regulation, as these bioinformatics searches also identified potential SigK binding sites upstream of the *ssp1* and *ssp4* genes of SM101. The presence of those SigK boxes could suggest that SigK, an alternative sigma factor, is also involved in the sporulation-associated regulation of some *ssp* genes. Further studies are underway to better understand how *C. perfringens* regulates Ssp expression.
